# Generative AI and student motivation: an SDT-guided systematic review of learning activities, motivational regulation, and measurement

**DOI:** 10.3389/fpsyg.2026.1984018

**Published:** 2026-10-28

**Authors:** Qi Pan, Hassan Abuhassna, Chuntao Jiang

**Affiliations:** 1 School of Foreign Languages, Zaozhuang University, Zaozhuang, China; 2 Department of Education, Faculty of Social Sciences and Liberal Arts, UCSI University, Kuala Lumpur, Malaysia; 3 Beizhuang Town Central Primary School, Zaozhuang, China

**Keywords:** basic psychological needs, generative artificial intelligence, self-determination theory, student motivation, systematic literature review

## Abstract

The application of Generative AI is reshaping students’ learning. However, student motivation in GenAI-supported learning is complex and influenced by multiple factors. To fully realize the educational potential of GenAI, a comprehensive understanding of the relationship between student motivation and GenAI is necessary. To address this gap, this study conducted a systematic literature review. From the perspective of Self-Determination Theory (SDT), this review examined 39 articles published between 2023 and 2025, drawn from two databases: Web of Science and Scopus. This study provides three major findings. First, competence-related learning activities were the most frequently identified across the reviewed studies. Second, four categories were reported in relation to student motivation: GenAI characteristics, learner factors, psychological factors, and environmental factors. Third, three major aspects of motivational conceptualization are basic psychological needs, motivation construct, and motivation-related construct. In addition, quantitative questionnaires were the dominant approach used to measure these constructs. Finally, the relationship among these three findings is illustrated. The findings provide insights into student motivation in GenAI-supported learning and highlight the relevance of GenAI characteristics, learners, and the learning environment. This study also provides theoretical and practical implications for facilitating students’ motivation in GenAI-supported learning environments.

## 1 Introduction

The rapid integration of Generative Artificial Intelligence (GenAI) into education has changed the ways students access information, receive feedback, and engage in learning (Qi P. et al., 2025). In this context, students’ motivation is particularly important, as students increasingly make decisions about how, when, and why they use GenAI. GenAI can enhance students’ learning experiences by providing personalized feedback, flexible learning pathways, and interactive communication (Beimel et al., 2025; Chen et al., 2025; Dou and Sun, 2025; Saleh and Alshewiter, 2025). However, the use of GenAI does not necessarily enhance students’ motivation to learn. Learners’ characteristics, self-regulated learning, and skills may also affect their active engagement in this learning environment. These different phenomena suggest that the relationship between GenAI and student motivation is complex. Therefore, how GenAI supports their motivation during learning remains an important question for effectively utilizing GenAI.

In GenAI-supported learning, features such as control over learning pace, feedback and practice, and interaction with GenAI, teachers, and peers may be relevant to students’ experiences of autonomy, competence, and relatedness. However, the presence of these features does not necessarily indicate that the corresponding psychological needs are satisfied. When these three basic psychological needs are satisfied, students’ motivation can be enhanced (Ryan and Deci, 2020). Therefore, SDT provides a theoretical basis for examining how different uses of GenAI may relate to students’ motivational experiences.

In recent years, research on GenAI and student motivation has grown rapidly. Existing studies have examined the effects of GenAI on autonomy, competence, relatedness, intrinsic motivation, engagement, self-efficacy, enjoyment, and other motivation-related constructs (Galindo-Domínguez et al., 2025; Ma and Chen, 2025; Wang and Wang, 2024). Besides these empirical studies, several reviews have identified the relationship between AI and student motivation from multiple perspectives (Table 1). For example, Li et al. (2026) and Huang et al. (2025) have synthesized motivational constructs, theories, interventions, and technological characteristics in AI- or chatbot-supported learning. Li et al. (2025b) focused on teacher roles in chatbot-assisted language learning from the SDT perspective. In the meantime, Qi et al. (2025a) examined the effects of GenAI on motivation and engagement. In addition, Qi et al. (2025b) also identified GenAI-supported learning activities and influencing factors. W. Wang et al. (2026) provide the motivational effects of AI-supported personalized feedback. These reviews demonstrate the growing scholarly attention to motivation in AI-supported learning.

**Table 1:** Past reviews on AI in student motivation.

No.	Author(s) (year)	Review type	Review focus
1	Li et al. (2026)	Scoping review	Student motivation in AI-supported learning: motivational constructs, theories, pedagogical approaches, and intervention duration
2	Huang et al. (2025)	Scoping review	Student motivation in chatbot-supported learning: motivational variables, theories, intervention designs, and chatbot characteristics
3	Li et al. (2025b)	Systematic literature review	Teacher roles in chatbot-assisted language learning from the SDT perspective
4	Qi et al. (2025a)	Systematic review and meta-analysis	Effects of GenAI on university students’ motivation and influencing factors
5	Qi et al. (2025b)	Systematic literature review	GenAI-supported SRL learning: pedagogical affordances, learning activities across SRL phases, and influencing factors
6	Wang W. et al. (2026)	Meta-analysis	Effects of AI-supported personalized feedback on students’ learning outcomes and motivation

Despite these contributions, several gaps remain. First, few reviews have examined what learning activities are in a GenAI learning environment. Second, few reviews have paid attention to systematically synthesizing how these learning activities support student motivation. Third, it remains unclear how student motivation has been conceptualized and measured across empirical studies on GenAI. It is important to understand further how and under what conditions these activities will lead to motivational support.

Therefore, the present study systematically reviews empirical research on student motivation in GenAI-supported learning from the perspective of SDT. Rather than focusing only on whether GenAI increases students’ motivation, we prefer to provide a more comprehensive understanding of GenAI-supported learning activities represented within an SDT theory, factors reported in relation to student motivation, and how motivation has been conceptualized and measured. Accordingly, the following research questions are proposed:

*RQ1*: What GenAI-supported learning activities are represented within an SDT-informed framework?

*RQ2*: What factors are reported in relation to student motivation in GenAI-supported learning?

*RQ3*: How has student motivation been conceptualized and measured in existing studies on Generative AI from the perspective of SDT?

## 2 Literature review

### 2.1 Self-Determination Theory (SDT)

According to Ryan and Deci (1985), Self-Determination Theory (SDT) emphasizes three core psychological needs: autonomy, competence, and relatedness. Autonomy refers to individuals’ need to feel in control of their own actions and decisions during the learning process. Competence is the perception of effectiveness and the ability to achieve learning goals. Relatedness represents the need to feel connected and maintain relationships with others in social contexts.

In educational contexts, when these three psychological needs are met, learners tend to achieve positive learning outcomes and personal development in the learning process (Liu et al., 2025; Ryan and Deci, 2020; Ryan et al., 2023), as the satisfaction of these psychological needs increases learners’ intrinsic motivation, which in turn promotes their deeper engagement and persistence in the learning process.

SDT not only posits the motivational processes but also provides an insightful perspective for understanding learners’ behaviors. This is closely related to digital learning environments, especially those supported by AI. AI has emerged as an innovative tool to support learners’ autonomy, competence, and relatedness by providing timely feedback, adaptive learning paths, and interactive communications (Adekoya et al., 2026; Mncube et al., 2026; Monzon and Hays, 2025). These features contribute to providing a supportive learning environment that fosters learners’ engagement and persistence in learning activities.

### 2.2 Generative AI and SDT

Generative AI is known as the subfield of Artificial Intelligence that creates new content and ideas, such as images, videos, audio, and software code, based on training data. The integration of generative AI has reshaped the educational context. For example, generative AI, like ChatGPT, can tailor learning content, adjust learning pace, and complexity to suit individual needs (Ayari and Ouerfelli, 2025; Jauhiainen and Garagorry Guerra, 2024; Sandhu et al., 2024). In addition, Cengiz et al. (2025) and Zhan and Yan (2025) found that the timely feedback of ChatGPT can enhance students’ engagement and writing proficiency (Cengiz et al., 2025; Zhan and Yan, 2025). Moreover, generative AI can improve learners’ sense of belonging, provide social support, and reduce loneliness (Henriksen et al., 2025; Kavitha et al., 2024). These findings suggest that Generative AI has the potential to support three psychological needs: autonomy, competence, and relatedness, as proposed by Self-Determination Theory by Ryan and Deci (1985).

From the perspective of autonomy, Generative AI technologies can enhance students’ autonomy in the learning process. For example, Generative AI provides adaptive learning paths, timely feedback, and intelligent tutoring systems; all of these features create a more personalized and autonomous learning environment, which enables students to regulate their own learning pace (Mncube et al., 2026). In terms of competence, Generative AI can improve students’ various competencies in learning. For instance, Generative AI encourages students to evaluate AI-generated content to promote students’ critical thinking and engagement (Pavlik, 2025). In addition, a generative AI- supported learning environment was demonstrated to improve students’ innovation (Wu and Zhang, 2025). Regarding relatedness, Generative AI can support students’ sense of social connection by offering an inclusive learning environment. For instance, Generative AI encourages diverse students to be involved in collaborative learning environments and reduces their learning anxiety by providing inclusive participation (Adekoya et al., 2026; Ala et al., 2025).

## 3 Methods

The present systematic literature review was conducted following the Preferred Reporting Items for Systematic Reviews and Meta-Analyses (PRISMA) guideline (Page et al., 2021). The review process consists of three stages: identification, screening, and inclusion (Qi and Abuhassna, 2026).

### 3.1 Literature selection

To ensure the comprehensive inclusion of relevant literature, Web of Science and Scopus were selected as the main data sources, as they were considered the major sources of bibliographic information in the academic field (Pranckutė, 2021). Moreover, the search query consists of three main dimensions: technical dimension (e.g., generative AI, ChatGPT); theoretical dimensions based on SDT (e.g., autonomy, competence, relatedness); outcome dimension (e.g., motivation, engagement, intrinsic motivation). To standardize the search process, similar terms were linked by the Boolean operator “OR,” while different dimensions were combined by “AND” (see Table 2).

**Table 2:** Databases and search queries.

Databases	Search queries
Scopus	TITLE-ABS-KEY ((“generative AI” OR “ChatGPT” OR “large language model*” OR “AI chatbot*” OR “artificial intelligence”) AND (“self-determination theory” OR autonomy OR competence OR relatedness) AND (motivation OR engagement OR “intrinsic motivation” OR “learning motivation” OR “student motivation”))
Web of Science	TS = ((“generative AI” OR “ChatGPT” OR “large language model*” OR “AI chatbot*” OR “artificial intelligence”) AND (“self-determination theory” OR autonomy OR competence OR relatedness) AND (motivation OR engagement OR “intrinsic motivation” OR “learning motivation” OR “student motivation”))

The literature search was conducted on 14th April, 2026. The search queries below (Table 2) were applied in Web of Science and Scopus. In Web of Science Core Collection, we selected the Topic field as a search term, yielding 1,142 articles. In Scopus, we chose the article title, abstract, and keywords as search terms, resulting in 1615 articles. In total, 2,757 records were identified for the screening stage (Figure 1).

**Figure 1:**
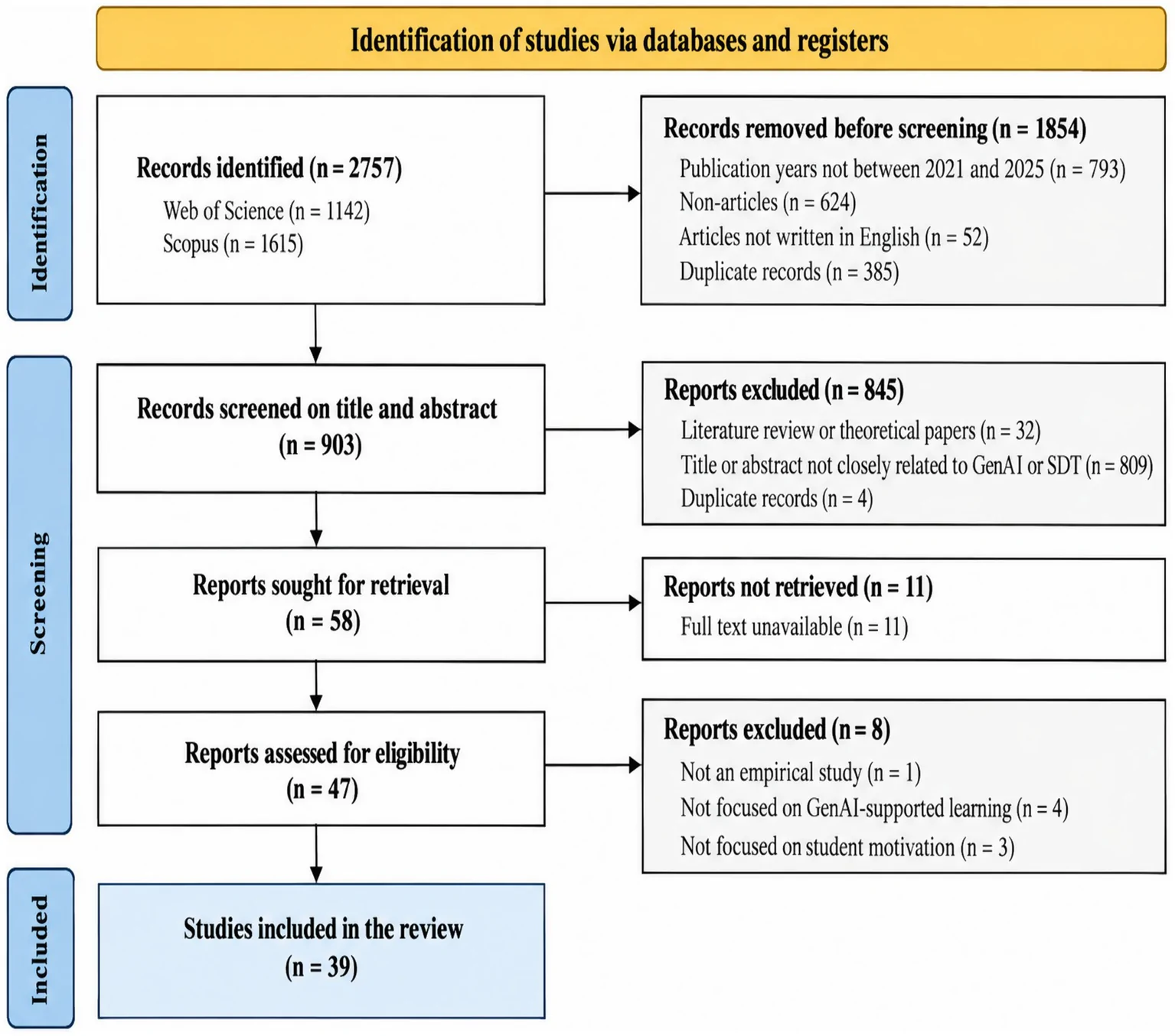
PRISMA flow diagram of the study selection process.

### 3.2 Literature screening and eligibility criteria

The literature selection and eligibility criteria are presented in Figure 1. The selection process involved three stages: initial identification of records, title and abstract screening, and full-text assessment. Two reviewers independently screened the titles, abstracts, and full texts according to the eligibility criteria. Disagreements were resolved through discussion until consensus was reached.

In the initial identification stage, we only selected articles published between 2021 and 2025, as the research on Gen AI in education has experienced a sharp development during this period (Ng and Ho, 2025; Zhu et al., 2025). In addition, only English journal articles were captured in this step. We removed articles not published between 2021 and 2025 (*n* = 793), non-articles (*n* = 624), duplicate articles (*n* = 385), and non-English articles (*n* = 52); see Table 3. As a result, 903 articles were left for the title and abstract screening.

**Table 3:** Eligibility criteria.

No.	Eligibility criteria	Description of criteria
1	Publication year	Articles were published between 2021 and 2025
2	Language	Articles were written in English
3	Research topic	Articles should focus on student motivation in GenAI-supported learning from an SDT perspective.
4	Research design	Empirical study

During the title and abstract screening stage, 845 articles were excluded due to the following reasons: literature reviews or theoretical papers (*n* = 32), unrelated to Gen AI or SDT (*n* = 809), and duplicate articles (*n* = 4). Consequently, 58 articles were sought for retrieval.

Of the 58 articles sought for retrieval, the full texts of 11 articles were unavailable. The remaining 47 articles were assessed for eligibility. We excluded articles based on the presented criteria: not empirical research (*n* = 1), not focused on GenAI-supported learning (*n* = 4), not focused on student motivation (*n* = 3). Details of the excluded studies and reasons for exclusion are provided in Supplementary Table 3. Finally, a total of 39 articles were included for the final analysis.

Development of analysis framework and coding process

Before literature coding, an analytical framework was developed to guide the coding process. Firstly, we adapted Self-Determination Theory (SDT) as a theoretical lens to examine students’ psychological needs, motivational regulation, and motivational constructs. In addition, we applied Chiu (2024) framework of 20 ChatGPT-supported learning activities to guide deductive coding of learning activities. These activities were originally classified into seven autonomy-related, eleven competence-related, and two relatedness-related activities. In the present study, these categories were used to code the learning activities identified in the included studies. The classification does not mean that these activities necessarily satisfy the corresponding psychological needs.

The coding process followed Braun and Clarke (2022) thematic analysis approach. First, study characteristics, such as author, publication year, region, research design, methodology, educational level, focus of study, and key findings were coded deductively. This coding approach provides a comprehensive overview of the characteristics of the studies included (Supplementary Table 1).

Subsequently, learning activities, factors related to student motivation in GenAI learning, conceptualization, and measurement of motivation were coded to address the research questions of this study. Learning activities were coded deductively based on the classifications of Chiu (2024). The descriptions of the 20 learning activities provided by Chiu (2024) were used to guide the coding. In addition, factors related to student motivation were coded inductively and then grouped into broader thematic categories. Motivational constructs and measures were coded as reported in the included studies.

The study was used as the unit of coding. Each study was counted only once for each code, although multiple codes could be assigned to the same study when more than one relevant activity, factor, or motivational construct was identified. Two coders independently coded the included studies. After independent coding, the results were compared by the two coders. Any discrepancies were resolved through discussions until a consensus was reached.

Given the heterogeneity of the included studies, a statistical meta-analysis was not conducted. Instead, a structured narrative synthesis was used. We considered these differences when interpreting the findings and distinguished experimental findings from cross-sectional associations and qualitative findings. The methodological quality of the included studies was also considered.

### 3.3 Methodological quality appraisal

The methodological quality of the included studies was assessed using the Mixed Methods Appraisal Tool (Hong et al., 2018). The MMAT was selected because it provides design-specific criteria for qualitative studies, randomized controlled trials, non-randomized studies, quantitative descriptive studies, and mixed-methods studies. Each study was first classified according to its research design and then assessed using the five MMAT criteria corresponding to that design. Two reviewers independently assessed the methodological quality of the included studies, and disagreements were resolved through discussion. Each criterion was rated as “Yes,” “No,” or “Cannot tell” based on the information reported in the original study. In accordance with the MMAT guidance, no overall numerical quality score was calculated. The detailed appraisal results are provided in the Supplementary Material.

## 4 Results

### 4.1 Descriptive statistics

The reviewed articles were published between 2023 and 2025, indicating the rapid growth of research on GenAI and students’ motivation in recent years. The characteristics of all reviewed studies were summarized in (Supplementary Table 1). Most of them used ChatGPT as a GenAI application to support student learning. Other GenAI applications included AI-supported gamification (Allehyani, 2025; Ma and Chen, 2025) and AI-driven personalized feedback (Xu et al., 2025). Furthermore, the reviewed articles were conducted across a wide range of countries and regions. Twenty-five out of thirty-nine articles were conducted in China. Moreover, the majority of studies focused on higher education (*n* = 34). These findings suggest that the current research is concentrated in Chinese and higher education contexts.

Considering research methodology, twenty-seven of the studies used a quantitative approach. Nine used mixed methods, and three adopted a qualitative approach. The included studies provided different types of evidence. Experimental and quasi-experimental studies generally reported changes in motivation or related outcomes, while cross-sectional studies mainly reported relationships between variables. Qualitative and mixed-methods studies reported both positive and negative experiences.

### 4.2 Methodological quality of the included studies

The MMAT appraisal indicated variation in the methodological quality of the included studies. Most studies met the criteria related to the appropriateness of measurements and data analysis. However, some methodological limitations were identified. In several quantitative studies, insufficient information was reported to determine the representativeness of the sample. Some randomized controlled trials did not provide sufficient information about blinding, while several mixed-methods studies provided limited information about the integration of quantitative and qualitative findings. Attrition was also a concern in one longitudinal study. Detailed criterion-level appraisal results are presented in Supplementary Table 2.

### 4.3 RQ1: what GenAI-supported learning activities are represented within an SDT-informed framework?

In this section, Chiu (2024)‘s classification of 20 GenAI-supported learning activities was applied as a framework to analyse the articles. Table 4 presents the coding results, while Figure 2 shows their distributions across three dimensions of SDT categories defined in Chiu (2024)‘s framework. Among these twenty learning activities, 14 were identified in the reviewed articles; however, six were not identified. In addition, learning activities classified under competence mostly appeared in the reviewed articles, with seven different types observed. Then, five types of learning activities were identified under autonomy and two under relatedness. The most frequently identified activities included getting feedback for students’ work, searching for information, getting examples, and getting insight into complex problems.

**Table 4:** Coding table of learning activities in GenAI-supported learning.

Learning activities	Autonomy	Competence	Relatedness	Count
1. Search for information	x			10
2. Get examples	x			10
3. Check their answers		x		5
4. Generate review questions to check for their understanding		x		0
5. Create new problems for practice		x		2
6. Create challenging problems		x		0
7. Get insight into complex problems	x			8
8. Ask ideas for their improvement		x		7
9. Make lists or outlines	x			3
10. Summarize their own work			x	4
11. Ask for definitions		x		5
12. Generate questions for discussions	x			2
13. Generate questions for essays	x			0
14. Get feedback for their work		x		17
15. Practice peer feedback		x		1
16. Prepare for tough conversations		x		2
17. Visualize a problem			x	1
18. Anticipate ChatGPT’s outputs		x		0
19. Grade ChatGPT’s outputs		x		0
20. Debate with ChatGPT	x			0

**Figure 2:**
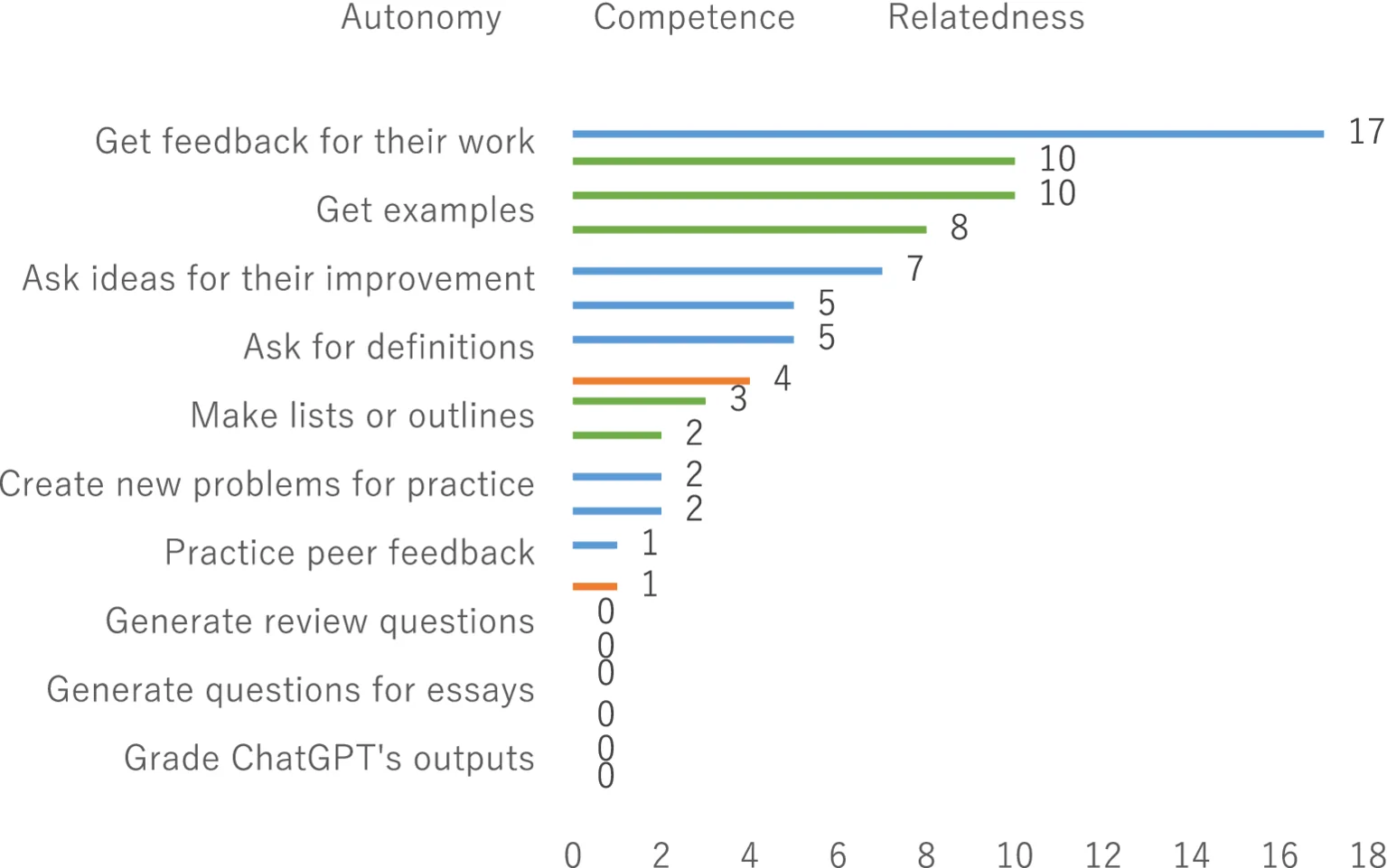
Distribution of GenAI-supported learning activities across the three SDT categories in Chiu (2024) framework.

In terms of the autonomy dimension, Figure 2 shows that learning activities mainly focused on information acquisition and knowledge exploration. According to Figure 2, searching for information, getting examples, and getting insight into complex problems were the most identified learning activities within this dimension. For information acquisition, students often used GenAI to generate examples for concept understanding (Annamalai et al., 2025; Hao et al., 2025; Saleh and Alshewiter, 2025). In the area of knowledge exploration, eight of the reviewed articles suggested that GenAI clarified difficult learning content and interpreted complex problems to deepen their understanding (Annamalai et al., 2025; Beimel et al., 2025; Hmoud et al., 2024; Wang and Wang, 2025).

These findings show that information acquisition and knowledge exploration were frequently identified among the activities in Chiu (2024) framework. However, the presence of these activities does not mean that students experienced greater autonomy. As illustrated in Figure 2, the competence dimension included the greatest number of learning activities. These learning activities were mainly represented through personalized feedback, learning improvement, and practice support.

1. Personalized feedback: A total of 17 articles highlighted the application of GenAI to provide personalized feedback during students’ learning process. The study by Allehyani (2025) pointed out that students received personalized AI feedback on their vocabulary and grammar learning to identify and correct their mistakes. The immediate and personalized feedback of GenAI was demonstrated to reduce students’ confusion and cognitive load, strengthen their competence and confidence, and finally enhance their motivation and long-term retention. Teng (2025) integrated GenAI into English writing instruction to support students’ motivation in the writing process. The results showed that personalized feedback from GenAI significantly improved students’ writing and their sustained motivation.
2. Learning improvement: Among the reviewed articles, learning improvement was usually supported by activities like asking for ideas for their improvement or asking for definitions, as these activities aimed to help learners refine their knowledge and deepen understanding. Research by Li et al. (2025a) shows that asking for improvement ideas from ChatGPT helps students refine their research ideas and academic writing, significantly enhancing their research skills. Similarly, research by Chen et al. (2025) also indicates that LLMs not only provide detailed explanations, example sentences, and translations but also help students understand lexical collocations and their contextual usage.
3. Practice support: Practice support not only provides students with opportunities to practice what they have learned, but also helps them apply knowledge in different learning contexts. Research by Hao et al. (2025) shows that an AI chatbot can provide students with personalized learning tasks to practice their acquired knowledge and enhance active vocabulary use. In addition, ChatGPT helps students prepare for discussions related to social interactions, which in turn reduces their social anxiety in real-life communication (Annamalai and Nasor, 2025).

The overall distribution shows that a variety of GenAI-supported learning activities were represented across the three SDT categories. However, some learning activities proposed in Chiu's (2024) framework were not identified in any reviewed study, such as generating review questions to check for their understanding, creating challenging problems, generating questions for essays, anticipating ChatGPT’s outputs, debating with ChatGPT, and grading ChatGPT’s outputs. These findings indicate several underexplored directions for future GenAI research.

Compared with autonomy and competence, only two learning activities were identified under relatedness in Chiu (2024)‘s framework: summarizing their own work and visualizing a problem. The reviewed studies reported these activities in relation to their summarizing skills, task completion (Hmoud et al., 2024), and visual comprehension (Allehyani, 2025). However, these studies did not directly examine whether these activities increased students’ relatedness. Considering this, we considered them as relatedness-related activities based on Chiu (2024)‘s framework.

### 4.4 RQ2: what factors are reported in relation to student motivation in GenAI-supported learning?

To address RQ2, we focused on the empirical studies included in this review (*n* = 39). We followed the thematic analysis approach proposed by Braun and Clarke (2022) and identified all factors relevant to motivation. As a result, a total of 35 factors were identified in the reviewed studies. The coding results were presented in Table 5.

**Table 5:** Factors reported in relation to student motivation in Gen AI learning environment.

No.	Factor	No. of studies
1	Personalized feedback	13
2	Perceived autonomy, competence and relatedness	12
3	Teacher support	9
4	Social connection	6
5	Self-regulated learning	5
6	Self-paced learning	4
7	Flexible learning opportunities	4
8	Human-like interaction	4
9	Emotional support	3
10	Non-judgmental learning environments	3
11	Cognitive overload	3
12	Peer interaction	3
13	English proficiency	2
14	AI literacy	2
15	Decision-making pressure	2
16	Enjoyment;	2
17	Institutional requirements	2
18	Learning progress monitoring	1
19	Error correction	1
20	Corrective feedback	1
21	Code Review	1
22	AI-mediated scaffolding	1
23	Supportive encouragement	1
24	Metacognitive strategy use	1
25	Digital competence	1
26	Student expertise	1
27	Self-efficacy	1
28	Resilience	1
29	Learning effort	1
30	Technical support	1
31	Academic assistance	1
32	Cultural factors	1
33	System quality	1
34	Information quality	1
35	Social anxiety reduction	1

According to Table 5, the most frequently reported factor is personalized feedback, which occurred 13 times across reviewed studies. Other frequently reported factors included perceived autonomy, competence, and relatedness (12), teacher support (9), social connection (6), self-regulated learning (5), self-paced learning (4), flexible learning opportunities (4), and human-like interaction (4). These frequencies indicate how often these factors were identified in the reviewed studies, rather than their importance or effect on student motivation. The factors were further classified into four categories according to their characteristics, such as GenAI characteristics, learner factors, psychological factors, and environmental factors.

#### 4.4.1 GenAI characteristics

GenAI characteristics were the most frequently identified category of factors in the reviewed studies. Through the analysis, we synthesized three major aspects, such as adaptive learning support, human-like interaction, and learner-centered design.

- Adaptive learning support: Adaptive learning support refers to assistance or resources provided by GenAI based on students’ needs. Unlike one-size-fits-all instruction, GenAI adjusts learning content, feedback, learning activities, and scaffolding to match students’ requirements. The reviewed articles indicated that adaptive learning support comprised: personalized feedback, learning progress monitoring, error detection and correction, and AI-mediated scaffolding. Previous studies have demonstrated that personalized feedback provided by GenAI improved students’ self-efficacy, learning engagement, and academic performance, which in turn enhanced their learning motivation (Chen et al., 2025; Wang et al., 2025; Xu et al., 2025). Furthermore, the study of Allehyani (2025) indicated that GenAI enhanced students’ motivation and long-term retention while reducing cognitive load through learning progress tracking and gamification features. Similarly, GenAI’s error detection and correction enable learners to identify their mistakes and receive immediate guidance. Such error detection and correction not only improved their learning performance but also enhanced their autonomy and competence (Choi and Kim, 2025; Du and Alm, 2024). Saleh and Alshewiter (2025) also highlighted the importance of AI-mediated learning scaffolding to enhance students’ digital competence, academic enjoyment, and engagement.
- Human-like interaction: Human-like interaction is about GenAI’s ability to communicate with learners. Studies by Hao et al. (2025) and Dou and Sun (2025) all pointed out that these human-like interactions involve emotional support, social connection, and supportive encouragement. It facilitated students’ metacognitive strategy use, digital competence, and learning engagement, thereby enhancing their learning motivation.
- Learner-centered design: Learner-centered design relates to instructional design of GenAI according to learners’ needs and preferences. In this study, learner-centered design across reviewed articles includes self-paced learning, flexible learning opportunities, and non-judgmental learning environments. The study by Beimel et al. (2025) and Hao et al. (2025) demonstrated that self-paced and flexible learning of GenAI favoured students’ learning experience, engagement, and motivation. In addition, the findings of Dou and Sun (2025) show that a non-judgmental learning environment provided by GenAI is a key factor in boosting students’ language acquisition, student motivation, psychological well-being (PWB), and psychological capital (PsyCap).

#### 4.4.2 Learner factors

Learner factors focus on individual characteristics reported in relation to student motivation in a GenAI learning environment. For example, previous studies pointed out that students’ readiness before using GenAI, such as English proficiency, AI literacy, digital literacy, and student expertise (Cao and Abdullah, 2025; Chiu et al., 2024; Liang and Reiss, 2025; Teng, 2025). In addition, students’ self-regulated learning and metacognitive strategies were also identified in the reviewed studies (Dou and Sun, 2025; Hao et al., 2025).

#### 4.4.3 Psychological factors

Psychological factors are another category reported in relation to student motivation in GenAI-assisted learning. Previous studies have demonstrated that basic psychological needs, including perceived autonomy, competence, and relatedness, were frequently reported across reviewed studies (Galindo-Domínguez et al., 2025; Shen and Cui, 2024; Singh and Aziz, 2025; Wang and Wang, 2025). In addition to these factors from SDT constructs, several studies also found learning enjoyment (Liang and Reiss, 2025), cognitive overload (Beimel et al., 2025), social anxiety reduction (Annamalai and Nasor, 2025), decision-making pressure (Wang and Wang, 2024), and resilience (Chen J., 2025) as factors influencing students’ motivation in such a learning environment.

#### 4.4.4 Environmental factors

Environmental factors refer to external conditions that influence students’ motivation in GenAI-assisted learning. We summarized this category into two main aspects: educational support and technical support.

- Educational support: Educational support refers to assistance or scaffolding provided by the learning environment, including teacher assistance and peer interaction. Previous studies have discussed the role of teacher assistance in relation to students’ basic psychological needs, expertise, and intention to learn with GenAI (Chen S., 2025; Chiu et al., 2024; Shen and Cui, 2024). Peer interaction was also reported in several studies. For example, Hsia et al. (2025) found that AI-supported Peer-Assisted Learning (AI-PAL) significantly improved students’ skills performance, learning motivation, autonomy, competence, and relatedness. Wang et al. (2025) also reported a relationship between peer interaction, students’ learning experience, engagement, and motivation.
- Technical support: Only a few discussed technical support in GenAI learning. System quality and information quality were reported in relation to students’ continuance intention towards GenAI-supported learning. In addition, Shen and Cui (2024) reported that technical support and teacher support were associated with students’ AI literacy through competence and autonomy.

### 4.5 RQ3: how has student motivation been conceptualized and measured in existing studies on generative AI from the perspective of SDT?

To address RQ3, we focused on how motivation and motivation-related constructs were conceptualized and measured in the reviewed studies. According to the results shown in Table 6, relevant constructs were organized into three major categories: basic psychological needs, motivation constructs, and motivation-related constructs. Basic psychological needs, including autonomy, competence, and relatedness, were frequently examined. Motivation constructs included intrinsic motivation, autonomous motivation, task motivation, and L2 writing motivation, as well as specific forms of motivational regulation, like identified regulation and controlled motivation. Other studies focused on motivation-related constructs, such as engagement, enjoyment, self-efficacy, and grit.

**Table 6:** Conceptualization and measurement of motivation and motivation-related constructs.

Category	Construct	Measurement examples	No. of studies (*n*)
Basic psychological needs	Autonomy, Competence, Relatedness	Basic Psychological Needs Scale (BPN Scale); Children’s Perceived Competence Scale, adolescent autonomy questionnaire, The Need for Relatedness Scale; Self-developed 17-item SDT-based Motivation Scale, Need Satisfaction Scale; Perceived autonomy scale; Semi-structured Interviews (SDT-based interview protocol)	17
Motivation constructs	Motivation	Motivation Questionnaire; Semi-structured Interviews, Classroom Observations, and LMS Logs; Motivation Scale	6
	Intrinsic Motivation	Motivation Questionnaire; Motivation Inventory (IMI); motivation scale; Intrinsic Motivation Scale	7
	L2 Writing Motivation	Motivation Scale; L2 Writing Motivation Scale	3
	Task Motivation	semi-structured individual interviews	1
	Motivation for Continuous Usage	Modified Self-Determination Theory questionnaire	1
	Autonomous motivation	Motivation Scale	2
	Identified Regulation	Self-developed 17-item SDT-based Motivation Scale	1
	Controlled Motivation	Motivation Scale	1
Motivation-related constructs	Academic enjoyment; Learning enjoyment	Academic Enjoyment Scale (AES); Learner Engagement Scale (LES)	2
	Behavioral engagement; Emotional engagement; Cognitive engagement	Learner Engagement Scale (LES); Student Engagement Questionnaire; The Utrecht Work Engagement Scale for Students; Flow State Scale (FSS-9)	10
	self-regulated learning	self-regulated learning scale	1
	self-efficacy; Writing Self-Efficacy	self-efficacy scale; Academic Self-Efficacy Questionnaire (ASEQ); Writing Self-Efficacy Scale	5
	GenAI-assisted writing engagement	GenAI-assisted writing engagement questionnaire	1
	learning desires	Desire for Learning (DFL) Scale	1
	future beliefs	Beliefs about the Future (BAF) Scale	1
	grit	Grit scale	2
	Writing Growth Mindset	Growth Mindset Scale	1
	Writing Interest	Writing Interest Scale	1
	Flow Experience	Flow Experience Scale	1

According to Tables 6, 17 studies examined three core components of basic psychological needs. Within SDT, these needs are theoretically related to motivation. Therefore, previous studies examined students’ autonomy, competence, and relatedness to understand students’ motivational experiences in a GenAI-supported learning environment. These constructs were often assessed using instruments like the Basic Psychological Needs Scale (BPN Scale), Children’s Perceived Competence Scale, adolescent autonomy questionnaire, Need for Relatedness Scale, Need Satisfaction Scale, and Perceived Autonomy Scale. Some studies also used self-developed measures or qualitative approaches, such as an SDT-based interview protocol.

The second category included studies that directly examined motivation or motivational regulation. These studies examined constructs like intrinsic motivation, L2 writing motivation, task motivation, autonomous motivation, and other forms of learning motivation, including identified regulation and controlled motivation. These constructs were assessed using general motivation questionnaires, the Intrinsic Motivation Inventory, and domain-specific motivation scales, like the L2 Writing Motivation Scale, and self-developed SDT-based measures.

The third category included motivation-related constructs. These constructs were associated with students’ motivational experiences. They included engagement, self-efficacy, enjoyment, grit, and so on. Among these constructs, engagement, such as emotional engagement, behavioral engagement, and cognitive engagement, was frequently examined (10 studies), followed by self-efficacy (5), enjoyment (2), and grit (2). Other constructs included self-regulated learning, GenAI-assisted writing engagement, learning desires, future beliefs, writing growth mindset, writing interest, and flow experience. The measurements of these studies refer to specific scales, such as the Learner Engagement Scale (LES), Academic Enjoyment Scale (AES), Self-Efficacy Scale, and Flow State Scale (FSS-9).

Based on the results above, we found that quantitative questionnaires represented the dominant approach to measuring motivation and related constructs. However, qualitative approaches, such as interviews, were less frequently employed. Different types of measurement were used in previous studies, such as established and self-developed measures.

## 5 Discussions

### 5.1 Synthesis and discussion of findings

This study conducted a systematic review of 39 articles to examine student motivation in GenAI-supported learning from the perspective of Self-Determination Theory (SDT). The results section presents three major findings. Figure 3 presents the relationship of these three findings. First, GenAI-supported learning activities were presented across the three SDT categories in Chiu (2024) framework. Second, diverse factors were reported in relation to student motivation. Third, motivation and motivation-related constructs were conceptualized in different ways and measured by multiple approaches. The findings should also be interpreted in light of differences in research design and methodological quality. Experimental and quasi-experimental studies provided evidence of changes in motivational or related outcomes, whereas cross-sectional studies primarily reported associations and qualitative studies reflected participants’ experiences and perceptions. The MMAT appraisal also identified methodological limitations in some studies, including limited information about sample representativeness, blinding, mixed-methods integration, and attrition. Therefore, the frequencies reported in this review indicate patterns across the literature rather than the strength of evidence, and findings based mainly on cross-sectional or methodologically limited studies should be interpreted cautiously.

**Figure 3:**
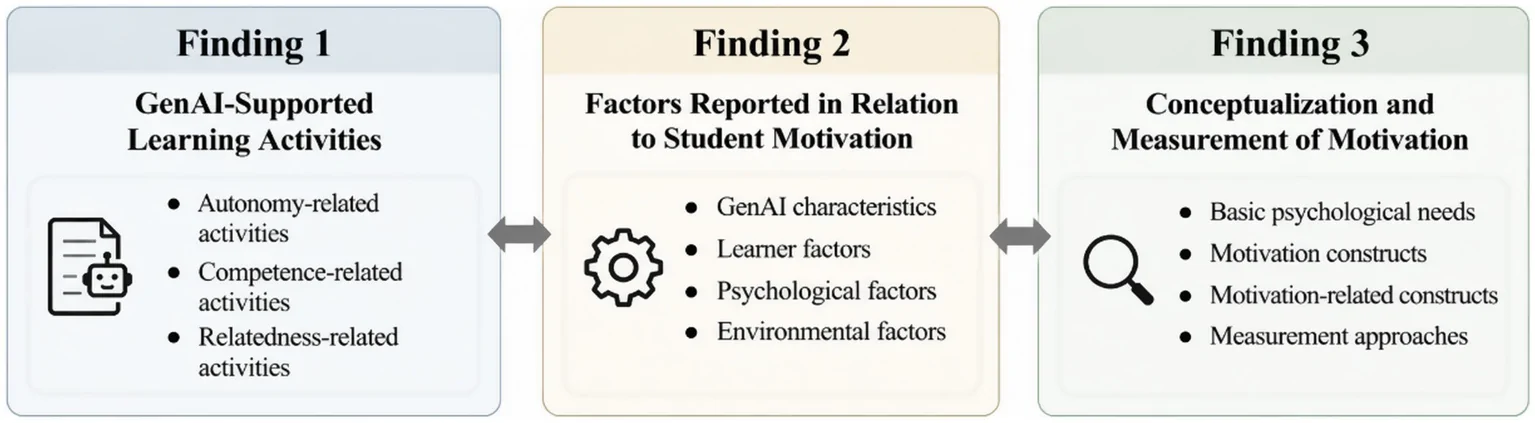
Relationships among the three major findings.

Our study builds on the systematic review by Qi et al. (2025b) with a specific focus on students’ motivation in GenAI from the perspective of SDT. Qi et al. (2025b) identified six pedagogical affordances and twenty student learning activities across the three phases of SRL to investigate how GenAI supports students’ SRL. They also identified individual and environmental factors influencing the effectiveness of GenAI-supported SRL. Notably, the individual factors included perceived autonomy, perceived competence, perceived relatedness, interest, and intrinsic motivation. We further examined which GenAI-supported learning activities were represented within an SDT-informed framework (Table 4), what factors were reported in relation to student motivation (Table 5), and how student motivation has been conceptualized and measured in existing studies (Table 6).

In the first finding, twenty learning activities proposed by Chiu (2024) were classified in relation to students’ autonomy, competence, and relatedness. However, according to the results presented in section 4.3, these three dimensions were not equally represented. Furthermore, competence-related learning activities were the most frequently identified. These activities refer to personalized feedback, learning improvement, and practice support. This finding suggests that current GenAI-supported learning focuses more on activities related to students’ competence. These learning activities help students improve their learning and experience a sense of mastery, which may further support their perceived competence (Dabbagh and Zgheib, 2026; Wang T. et al., 2026). However, the presence of these activities does not indicate that students’ competence needs were satisfied.

However, six learning activities were not identified in the reviewed studies. These included generating review questions to check for their understanding, creating challenging problems, generating questions for essays, anticipating ChatGPT’s outputs, grading ChatGPT’s outputs, and debating with ChatGPT. This finding is consistent with previous studies showing that current uses of GenAI focus more on receiving information, feedback, and learning support, whereas learning activities that require students to evaluate, question, or challenge GenAI outputs have received less attention (Galdames and Chaple-Gil, 2026; López-López et al., 2026).

The reviewed studies paid limited attention to need frustration and how GenAI may affect students’ autonomy, competence, or relatedness. Future studies should examine need satisfaction, as well as need frustration in GenAI-supported learning.

The second finding identified four categories of factors reported in relation to student motivation, such as GenAI characteristics, learner factors, psychological factors, and environmental factors. Among them, GenAI characteristics were the most frequently identified. This finding suggests that technological characteristics are commonly examined in relation to students’ motivational experiences in GenAI-supported learning. These factors, such as personalized feedback, flexible learning opportunities, and human-like interaction, may provide conditions that support students’ competence, relatedness, and autonomy. However, the presence of these characteristics does not necessarily lead to stronger motivation.

Learner factors included students’ readiness, such as English proficiency, AI literacy, digital literacy, and student expertise; self-regulated learning; and metacognitive strategy use. These findings suggest that learners’ knowledge, skills, and learning strategies are also related to their motivational experiences in Gen AI learning. Students with different levels of knowledge and skills may have different experiences when using GenAI tools (Chiu et al., 2024). For example, students with limited AI literacy may have difficulty asking appropriate questions or evaluating AI-generated information. Therefore, sufficient knowledge and skills may help students to use GenAI more effectively.

Besides that, psychological factors were also identified in this review. Perceived autonomy, competence, and relatedness were frequently reported. This finding highlights the relevance of students’ psychological experiences in GenAI. Students’ motivation may therefore be related not only to technical features provided by GenAI, but also to students’ psychological experience when using it (Schweder et al., 2026). In addition, environmental factors were also reported in relation to student motivation in GenAI-supported learning, particularly teacher support and peer interaction. Teachers are still needed to guide students in using GenAI (Li and Chiu, 2025). Peer interaction also provides opportunities for communication and social support. In conclusion, student motivation was related to technological, learner, psychological, and environmental factors, rather than GenAI characteristics alone (Syukur et al., 2025; Tamura et al., 2023).

The third finding identified basic psychological needs, motivation constructs, and motivation-related constructs. Basic psychological needs focus on autonomy, competence, and relatedness, while motivation-related constructs such as engagement, self-efficacy, enjoyment, and grit are related to motivation. Although the reviewed studies also paid attention to specific forms of motivational regulation, like autonomous motivation, identified regulation, and controlled motivation, these constructs were less frequently investigated compared to other constructs. This is important because motivational regulation concerns why students are motivated or the quality of their motivation. Students may engage in learning for different reasons, from controlled to more autonomous motivation (Ryan and Deci, 2020).

In terms of measurement, quantitative questionnaires were the dominant approach. The reviewed studies used different types of measurement, including established and self-developed measures. Besides that, student motivation in GenAI-supported learning may vary according to learning tasks, interaction, and the support. In this way, quantitative questionnaires may not fully capture the development and changes of student motivation during their actual learning within GenAI. Therefore, qualitative approaches, such as interviews, learning diaries, observations, and GenAI interaction data, may provide a more comprehensive understanding of students’ learning experiences and their motivational development within a GenAI learning environment. This review focused on how motivation was conceptualized and measured in the included studies. However, the validity and reliability of the instruments were not further examined. Whether these instruments were based on SDT or suitable for different research contexts was also not specifically analyzed.

### 5.2 Limitations

This review has several limitations. First, most of the included studies were conducted in China (25 of 39) and in higher education (34 of 39), which may limit the generalizability of the findings to other educational and cultural contexts. Second, the included studies varied in research design, GenAI use, and motivational measurement, which makes direct comparison across studies difficult. Therefore, the findings should be interpreted with these differences in mind. Third, a formal inter-coder agreement statistic was not calculated during the original coding process, and the pre-consensus coding records were not retained. The reliability of the coding could not be independently assessed using an inter-coder agreement coefficient. Fourth, the search strategy also has some limitations. The use of SDT-related terms may have excluded relevant studies that examined student motivation using different terms. In addition, the broad term “artificial intelligence” may have resulted in the retrieval of studies that were not directly related to GenAI. Therefore, the findings of this review are limited to the studies identified through this search strategy.

### 5.3 Future directions

Based on the findings of the present literature review, we propose four major future directions.

*More research is needed on learning activities using GenAI*. Current studies mainly focus on using GenAI to provide information, feedback, and learning support. To deepen understanding of different GenAI-supported learning activities related to students’ autonomy, competence, and relatedness, future research could pay more attention to activities involving questioning, reflection, and evaluation.

*More research is needed from a human-AI collaborative perspective*. The findings of this review identified multiple factors related to students’ motivation in GenAI-supported learning, from technical features to human-related features. Future research could investigate how human–AI collaboration is related tostudents’ motivational experiences during learning.

*More research is needed on motivational regulation in GenAI-supported learning*. Existing studies have primarily examined basic psychological needs, motivation, and motivation-related constructs. However, limited attention has been paid to motivational regulation. Future research could further investigate why students engage in GenAI-supported learning and how different types of motivation develop during their learning with GenAI.

*More research is needed, combining diverse methodological approaches*. Quantitative questionnaires were the dominant approach in the reviewed studies. Future research should combine quantitative questionnaires with qualitative and longitudinal approaches to provide a more comprehensive understanding of student motivational experiences and their development during GenAI-supported learning.

## 6 Conclusion

This systematic literature review provides a comprehensive insight into students’ motivation in a GenAI learning environment from the perspective of SDT. The findings suggest that student motivation within GenAI-supported learning is related not only to technological characteristics but also to how students use and interact with GenAI tools. Specifically, this review has two implications below:

1) Theoretical implications:

From a theoretical perspective, this review examined GenAI-supported learning activities, factors related to student motivation, as well as the conceptualization of students’ motivation. The findings extend the understanding of GenAI-supported motivation and highlight the need to consider the quality of students’ motivation.

2) Practical implications:

The findings offer some practical suggestions. First, educators should carefully design learning activities that promote higher-order thinking. For instance, educators should encourage students to actively interact with, question, and evaluate GenAI, not simply to receive feedback. Second, educators should provide appropriate guidance and facilitate peer interaction during the learning process. Third, students need to know how to use GenAI effectively and appropriately. Therefore, educators should develop students’ readiness to use GenAI, particularly their AI literacy, self-regulated learning, and metacognitive skills.
