# Supplementary material for: Generative AI and student motivation: an SDT-guided systematic review of learning activities, motivational regulation, and measurement
Source: Front Psychol. 2026 Oct 28;17:1984018. doi: 10.3389/fpsyg.2026.1984018 (PMC13751964; doi:10.3389/fpsyg.2026.1984018)
Supplement: Supplementary file 1 [file Data_Sheet_1.docx]

Supplementary Table 1: Summary of the included articles

| ID | Author (year) | Region | Research design | Sample/Level | GenAI use/ intervention | Key findings |
| --- | --- | --- | --- | --- | --- | --- |
| A1 | Shoeb Saleh, Kosay Moneer Alshewiter（2025） | Saudi Arabia | Quasi-experimental design | 140 university EFL learners | Conversational AI; 8 curriculum units | AI intervention significantly improved students' digital competence, academic enjoyment, and engagement compared with conventional instruction. |
| A2 | Hao et al. (2025) | China | Quasi-experimental | 103 Chinese private-college students | LLM chatbot; 8 weeks | AI chatbot significantly improved vocabulary performance. Perceived autonomy was the strongest motivational factor, and metacognitive strategy use mediated the effect of autonomy on vocabulary outcomes. |
| A3 | Faris Salim Allehyani （2025） | Saudi  Arabia | Experimental Mixed-Methods | 85 Saudi EFL learners | AI-powered gamification; 6 weeks | AI-powered gamification significantly enhanced learners’ motivation and long-term retention, while reducing cognitive load. |
| A4 | Annamalai et al.(2025) | Malaysia | Cross-sectional survey design | 324 university EFL/ESL students | ChatGPT; prior use | Findings show that autonomy is the most important and highest factor determining students’ motivation for continuous usage of ChatGPT. |
| A5 | Weiwei Dou et al.(2025) | China | Quasi-experimental design | 158 university EFL learners | ChatGPT, Kimi, Doubao; 4 weeks | Findings demonstrated a significant positive effect of ChatGPT on EFL learners’ motivation, PsyCap, and PWB |
| A6 | Galindo-Domínguez et al.(2025) | Spain | Longitudinal Intervention Study design | 50 secondary students | AI subject; 7 months | Findings show that only relatedness improved significantly after the AI intervention. Competence was the strongest predictor of intrinsic motivation, and initial motivation predicted motivation over time. |
| A7 | Lei Cao et al.(2025) | China | Case study | 39 vocational-college EFL students | Xuexitong, Doubao, DeepSeek, Kimi; two semesters | Findings demonstrated that students who engage behaviorally with GenAI tools lack intrinsic motivation and emotional connection. |
| A8 | Dizza Beimel et al.(2025) | Israel | Controlled experimental  design | 96 participants; 72 complete cases; university | GenAI tool of choice; main experimental session ~75 min | AI-enhanced learning experience and efficiency, but instructor-led sessions remained critical for deep engagement and complex problem-solving. |
| A9 | Lei Shen et al.(2025) | China | cross-sectional design | 364 undergraduates + 29 teachers | GenAI-assisted writing | The LPA identified three engagement profiles (high, medium, and low). The one-way ANOVA demonstrated that there was no significant relationship between GenAI attitude and writing engagement. Teachers perceived GenAI as beneficial for enhancing behavioral, emotional, and cognitive engagement. |
| A10 | Ziyi Gao et al.(2024) | China | Cross-sectional Survey Design | Chinese university business students | ChatGPT | ChatGPT contributed to better academic performance by increasing learning desire and future beliefs. However, self-efficacy had no significant relationship with academic performance. |
| A11 | Mark Feng Teng(2025) | China | Longitudinal survey design | 261 first-year university students | Poe GenAI feedback; one academic year | GenAI significantly enhanced EFL learners’ autonomy, competence, relatedness, and identified regulation. The development of Motivation followed a curvilinear trajectory. Moreover, English proficiency positively predicted motivational development. |
| A12 | Jingjing Chen(2025) | China | Cross-sectional survey design | 382 university Chinese-language learners | AI-driven learning context | Grit positively predicted self-efficacy and engagement; self-efficacy directly predicted engagement. |
| A13 | Nagaletchimee Annamalai et al(2025) | United Arab Emirates | Cross-sectional Mixed Methods Design | 83 undergraduates + 20 interviewees | ChatGPT; prior academic use | ChatGPT supported learners’ competence, relatedness, and autonomy. However, participants reported a lack of emotional and human connection. |
| A14 | Wang et al(2024) | China | Cross-sectional mixed-methods design | 840 Chinese university students | AI-supported English learning | Most BPNs played a critical role in predicting behavioral, cognitive, and emotional engagement. However, perceived autonomy did not predict behavioral engagement, and perceived competence did not predict behavioral or cognitive engagement. |
| A15 | Ziwen Pan et al(2025) | China | Cross-sectional Survey Design | 627 Chinese HE students | AI-supported EFL writing | Teacher affective support and AI literacy significantly predicted writing self-efficacy and L2 writing motivation. In addition, writing self-efficacy also directly predicted motivation and mediated teacher affective support and AI literacy |
| A16 | Tummalapenta et al(2025) | India | Cross-sectional Survey Design | 673 Indian HE students reported in paper | ChatGPT; most had prior use, many 3–6 months | Autonomy and relatedness significantly affected perceived usefulness and perceived ease of use. However, perceived competence did not significantly predict perceived usefulness. |
| A17 | Jinqiao Zhou et al (2024) | China | Cross-sectional survey design | 387 Chinese university students | AI tools for self-directed learning | Information Quality, System Quality, Perceived Competence, and Intrinsic Motivation significantly influenced continuance intention toward AI-supported SDL. |
| A18 | Mohammad Hmoud et al(2024) | Palestine | Qualitative Research Design | 15 graduate students | ChatGPT 3.5; ≥1 semester prior experience | ChatGPT positively enhanced students’ task motivation into five dimensions, including task enjoyment, reported effort, result assessment, perceived relevance, and interaction |
| A19 | Yajing Fu et al(2025) | China | Longitudinal Study | 313 complete cases from 837 at T1; university | Kimi, Doubao, DeepSeek; three waves over ~6 months | Autonomy, competence, and relatedness showed significant growth over time. Autonomy increased the most. In the meantime, autonomy and relatedness showed synchronized developmental trajectories. |
| A20 | Harman Preet Singh et al(2025) | Saudi  Arabia | Cross-sectional Survey Design | 304 Saudi undergraduates | AI/Intelligent Learning Assistants | Autonomy, competence, and relatedness all positively predicted creativity. However, autonomy had the strongest effect. |
| A21 | Chen et al (2025) | China | Randomized Controlled Trial (RCT) | 70 Chinese sophomores | Coze-based CBLP-LLM; 5 weeks + 1-month follow-up | Students' writing motivation was improved significantly, while reducing cognitive load. |
| A22 | Qi An(2025) | China | Cross-sectional Survey Design | 509 HE students | AI-assisted EFL learning | Autonomy, competence, and relatedness significantly predicted learners’ grit, while grit positively influenced engagement. Finally, basic psychological needs had a direct effect on engagement. |
| A23 | Jingjing Chen(2025) | China | Cross-sectional Survey Design | 630 Chinese undergraduates | General AI-enhanced CSL context | CSL learners’ motivation, engagement, and resilience in AI-enhanced contexts are interrelated, particularly the resilience of learners  is the strongest predictor of their engagement |
| A24 | Yanyan Shen et al(2024) | China | Cross-sectional Survey Design | 445 university students | Multiple AI-education systems/tools | Teacher support and technical support positively predicted BPN. However, autonomy and competence significantly predicted AI literacy and mediated the relationship between support and AI literacy. |
| A25 | Shujiao Chen(2025) | China | Cross-sectional Survey Design | 442 international students in Chinese HE | AI-enhanced learning environments | Perceived teacher support positively predicted motivation, engagement, and PWB. In addition, motivation significantly mediated the relationship between teacher support and both engagement and psychological well-being. |
| A26 | Lu-Ho Hsia et al(2025) | China | Quasi-experimental Design | 90 technological-university students | AI-supported peer-assisted learning | AI-PAL significantly improved students' skills performance, learning motivation, autonomy, competence, and relatedness compared with conventional PAL |
| A27 | Seongyune Choi et al (2024) | South Korea | Quasi-experimental Design | 215 first-year high-school students | LLM-based programming learning environment | LPLE significantly improved students' programming ability, autonomous motivation, autonomy, competence, and relatedness. However, no significant improvement was found for controlled motivation. |
| A28 | Hao Tranet al(2025) | Australia and Vietnam | Cross-sectional Survey Design | 416 survey + 17 interviews; Australia/Vietnam HE | Machine translation + GenAI for academic writing | The findings reported moderate levels of autonomy, competence, and relatedness in using MT/GAI. In addition, the ESL/EFL context significantly influenced relatedness, and disciplinary differences also affected students’ perceptions. In the meantime, teacher and peer support, as well as institutional policies, also shaped students’ experiences. |
| A29 | Thomas K.F. Chiu et al(2023) | China | Quasi-experimental Design | 123 Grade 10 students | AI chatbot + varying teacher support | Teacher support moderated the relationships between student expertise, students' basic need satisfaction, and intrinsic motivation. Teacher support strengthened relatedness satisfaction but reduced autonomy satisfaction. |
| A30 | Liang and Reiss (2025) | China | Two-wave survey design | 425 university students | General AI use | Positive attitudes significantly enhanced learning engagement. Autonomy and learning enjoyment mediate this relationship. |
| A31 | Ma et al(2025) | China | Longitudinal quasi-experimental design | 150 university EFL learners | LinguaQuest AI ± teacher scaffolding; 14-week intervention, delayed test | The AI + Scaffolding group achieved significantly greater proficiency gains than the AI Only and Control groups. In addition, the satisfaction of SDT needs significantly mediates motivation. Finally, Teacher scaffolding played a pivotal role in contextualizing AI feedback. |
| A32 | Yi Li et al(2025) | Pakistan | Quasi-experimental research design | 366 final-year undergraduates | ChatGPT 3.5-integrated instruction; 10 weeks | ChatGPT-based instruction significantly improved students' research skills, motivation,  engagement, and self-directed learning. |
| A33 | Caili Wang et al(2025) | China | Quasi-experimental design | 94 Chinese undergraduates | ChatGPT / Grammarly; 15 one-hour sessions | ChatGPT and Grammarly significantly improved students' emotional regulation, motivation, psychological well-being, and academic writing compared with traditional instruction. |
| A34 | Jinming Du et al(2024) | New Zealand. | Qualitative Research Design | 24 postgraduate EAP students | ChatGPT | ChatGPT supported students’ autonomy and competence. However, its impact on relatedness was mixed, |
| A35 | Qunai Xu et al (2025) | China | Cross-sectional survey | 1,079 Chinese HE students | AI-driven personalized feedback | AI-driven personalized feedback significantly and positively predicted goal achievement, self-efficacy, and learning engagement. |
| A36 | Wu, Li et al (2025) | China | Sequential explanatory design | 506 survey + 18 interviews; Chinese EFL learners | GenAI chatbots | Perceived support significantly predicted BPNs. BPNs positively influenced engagement, and competence mediated all four engagement dimensions. |
| A37 | Xiaochen Wang et al (2025) | China | Mixed-methods design | 210 Chinese university EFL learners | LLMs such as ChatGPT | Perceived relatedness predicted behavioral, cognitive, and emotional engagement. Perceived autonomy predicted cognitive and emotional engagement. Perceived competence predicted emotional engagement. |
| A38 | Surachmi et al. (2025) | Indonesia | Cross-sectional survey design | 462 EFL undergraduate students / Higher education | GenAI for English learning | Students’ autonomy and relatedness were associated with their intention to use GenAI for English learning. Autonomy, competence, and relatedness were also associated with their actual use of GenAI. |
| A39 | Zhou & Ma (2025) | China | Cross-sectional survey design | 669 university students / Higher education | GenAI-assisted informal digital English learning (GAI-IDLE) | Control and flow experience were associated with students’ continued use of GenAI for informal English learning. |

Supplementary Table 2. Methodological Quality Appraisal of the Included Studies Using the MMAT

| **Study** | **Study design** | **C1** | **C2** | **C3** | **C4** | **C5** |
| --- | --- | --- | --- | --- | --- | --- |
| A1 | Quantitative non-randomized | CT | Y | Y | Y | Y |
| A2 | Quantitative randomized controlled trial | Y | Y | Y | CT | Y |
| A3 | Mixed methods | Y | Y | Y | Y | Y |
| A4 | Quantitative descriptive | Y | CT | Y | Y | Y |
| A5 | Quantitative non-randomized | CT | Y | Y | Y | Y |
| A6 | Quantitative non-randomized | N | Y | Y | CT | Y |
| A7 | Qualitative | Y | Y | Y | Y | Y |
| A8 | Mixed methods | Y | Y | Y | CT | Y |
| A9 | Mixed methods | Y | Y | Y | Y | Y |
| A10 | Quantitative descriptive | Y | CT | Y | Y | Y |
| A11 | Quantitative randomized controlled trial | Y | CT | Y | CT | Y |
| A12 | Quantitative descriptive | CT | CT | Y | Y | Y |
| A13 | Mixed methods | Y | Y | Y | Y | Y |
| A14 | Quantitative descriptive | CT | CT | Y | Y | Y |
| A15 | Quantitative descriptive | CT | CT | Y | Y | Y |
| A16 | Quantitative descriptive | CT | CT | Y | CT | Y |
| A17 | Quantitative descriptive | CT | CT | Y | Y | Y |
| A18 | Qualitative | Y | Y | Y | Y | Y |
| A19 | Quantitative non-randomized/longitudinal | CT | Y | N | CT | Y |
| A20 | Quantitative descriptive | CT | CT | Y | Y | Y |
| A21 | Randomized controlled trial | Y | Y | Y | CT | Y |
| A22 | Quantitative descriptive | CT | CT | Y | Y | Y |
| A23 | Quantitative descriptive | CT | CT | Y | Y | Y |
| A24 | Quantitative descriptive | CT | CT | Y | Y | Y |
| A25 | Quantitative descriptive | CT | CT | Y | Y | Y |
| A26 | Quantitative non-randomized | CT | Y | Y | Y | Y |
| A27 | Mixed methods | Y | Y | Y | CT | Y |
| A28 | Mixed methods | Y | Y | Y | Y | Y |
| A29 | Quantitative randomized controlled trial | Y | CT | Y | CT | Y |
| A30 | Quantitative descriptive | CT | CT | Y | Y | Y |
| A31 | Mixed methods | Y | Y | Y | Y | Y |
| A32 | Quantitative non-randomized | CT | Y | Y | Y | Y |
| A33 | Quantitative non-randomized | CT | Y | Y | Y | Y |
| A34 | Qualitative | Y | Y | Y | Y | CT |
| A35 | Quantitative descriptive | N | CT | Y | Y | Y |
| A36 | Mixed methods | Y | Y | Y | Y | Y |
| A37 | Mixed methods | Y | Y | Y | CT | Y |
| A38 | Quantitative descriptive | Y | CT | Y | Y | Y |
| A39 | Quantitative descriptive | Y | CT | Y | Y | Y |

**Note.** The appraisal criteria were based on the design-specific criteria of the Mixed Methods Appraisal Tool (MMAT; Hong et al., 2018). Y = Yes; N = No; CT = Can’t tell. The meaning of C1–C5 varies according to study design, as follows:

**Qualitative studies:** C1 = appropriateness of the qualitative approach; C2 = adequacy of qualitative data collection; C3 = adequate derivation of findings from the data; C4 = substantiation of the interpretation by the data; C5 = coherence among data sources, data collection, analysis, and interpretation.

**Randomized controlled trials:** C1 = appropriate randomization; C2 = baseline comparability of groups; C3 = completeness of outcome data; C4 = blinding of outcome assessors; C5 = adherence to the assigned intervention.

**Quantitative non-randomized studies:** C1 = representativeness of participants; C2 = appropriateness of measurements of the outcome and intervention/exposure; C3 = completeness of outcome data; C4 = consideration of confounders in the design and analysis; C5 = administration of the intervention/exposure as intended.

**Quantitative descriptive studies:** C1 = relevance of the sampling strategy; C2 = representativeness of the sample; C3 = appropriateness of measurements; C4 = low risk of nonresponse bias; C5 = appropriateness of statistical analysis.

**Mixed-methods studies:** C1 = adequate rationale for using a mixed-methods design; C2 = effective integration of qualitative and quantitative components; C3 = adequate interpretation of the integrated findings; C4 = adequate consideration of divergences and inconsistencies between quantitative and qualitative results; C5 = adherence of the different components to the quality criteria of their respective methodological tradition

Supplementary Table 3: Excluded Studies and Reasons for Exclusion

| **No.** | **Study** | **Reason for exclusion** | **DOI** |
| --- | --- | --- | --- |
| 1 | Huber et al. (2024), *Leveraging the Potential of Large Language Models in Education Through Playful and Game-Based Learning* | Not an empirical study | https://doi.org/10.1007/s10648-024-09868-z |
| 2 | Şimşek et al. (2025), *The predictive effect of nursing students’ attitudes and acceptance towards artificial intelligence on their clinical competencies* | Not focused on student motivation | <https://doi.org/10.1016/j.teln.2025.02.036> |
| 3 | Sari & Han (2024), *The impact of automated writing evaluation on English as a foreign language learners’ writing self-efficacy, self-regulation, anxiety, and performance* | Not focused on GenAI-supported learning | <https://doi.org/10.1111/jcal.13004> |
| 4 | Chai et al. (2023), *Modeling Chinese Secondary School Students’ Behavioral Intentions to Learn Artificial Intelligence with the Theory of Planned Behavior and Self-Determination Theory* | Not focused on GenAI-supported learning | <https://doi.org/10.3390/su15010605> |
| 5 | Wang, Cui, & Yuan (2025), *Artificial Intelligence in Higher Education: The Impact of Need Satisfaction on Artificial Intelligence Literacy Mediated by Self-Regulated Learning Strategies* | Not focused on GenAI-supported learning | <https://doi.org/10.3390/bs15020165> |
| 6 | Liu (2025), *Profiles of AI anxiety among Chinese public administration students and the predictive role of resilience: A self-determination theory perspective* | Not focused on student motivation | [10.1016/j.lmot.2025.102207](https://doi.org/10.1016/j.lmot.2025.102207) |
| 7 | Pan & Li (2025), *The effects of perceived teacher support and growth language mindset on learner well-being in AI-integrated environment: the mediating role of generative AI attitude* | Not focused on student motivation | <https://doi.org/10.3389/fpsyg.2025.1660462> |
| 8 | Ode et al. (2025) A cross-country analysis of self-determination and continuance use intention of AI tools in business education: Does instructor support matter? | Not focused on GenAI-supported learning | [10.1016/j.caeai.2025.100402](https://doi.org/10.1016/j.caeai.2025.100402) |
